# Animal Cell Differentiation Patterns Suppress Somatic Evolution

**DOI:** 10.1371/journal.pcbi.0030250

**Published:** 2007-12-14

**Authors:** John W Pepper, Kathleen Sprouffske, Carlo C Maley

**Affiliations:** 1 Department of Ecology and Evolutionary Biology, University of Arizona, Tucson, Arizona, United States of America; 2 The Santa Fe Institute, Santa Fe, New Mexico, United States of America; 3 Genomics and Computational Biology Program, University of Pennsylvania, Philadelphia, Pennsylvania, United States of America; 4 Systems Biology Division, The Wistar Institute, Philadelphia, Pennsylvania, United States of America; University of Texas at Austin, United States of America

## Abstract

Cell differentiation in multicellular organisms has the obvious function during development of creating new cell types. However, in long-lived organisms with extensive cell turnover, cell differentiation often continues after new cell types are no longer needed or produced. Here, we address the question of why this is true. It is believed that multicellular organisms could not have arisen or been evolutionarily stable without possessing mechanisms to suppress somatic selection among cells within organisms, which would otherwise disrupt organismal integrity. Here, we propose that one such mechanism is a specific pattern of ongoing cell differentiation commonly found in metazoans with cell turnover, which we call “serial differentiation.” This pattern involves a sequence of differentiation stages, starting with self-renewing somatic stem cells and proceeding through several (non–self-renewing) transient amplifying cell stages before ending with terminally differentiated cells. To test the hypothesis that serial differentiation can suppress somatic evolution, we used an agent-based computer simulation of cell population dynamics and evolution within tissues. The results indicate that, relative to other, simpler patterns, tissues organized into serial differentiation experience lower rates of detrimental cell-level evolution. Self-renewing cell populations are susceptible to somatic evolution, while those that are not self-renewing are not. We find that a mutation disrupting differentiation can create a new self-renewing cell population that is vulnerable to somatic evolution. These results are relevant not only to understanding the evolutionary origins of multicellularity, but also the causes of pathologies such as cancer and senescence in extant metazoans, including humans.

## Author Summary

Darwinian natural selection and evolution is usually studied in populations of organisms. However, it is possible, in principle, in any population of cells, including the population of cells that constitutes a multicellular animal. Such “somatic” evolution among cells within an organism tends to reduce their cooperation, and thus to threaten the integrity of the organism. It is believed that this problem must have been solved somehow to allow the evolutionary emergence of multicellular animals. However, it has also been suggested that some pervasive pathologies reflect the persistence of some level of somatic evolution Here, we propose that a well-known pattern of ongoing cell differentiation in the mature tissues of animals functions to suppress somatic evolution. We test his hypothesis using a computer simulation of cell population dynamics and evolution. The results are consistent with our hypothesis, and suggest that cancer and senescent decline with aging may be attributable to a failure of this mechanism to completely suppress cellular evolution.

## Introduction

### The Puzzle of Ongoing Cell Differentiation

Mature tissues of long-lived metazoans exhibit ongoing cell differentiation, with tissue-specific somatic stem cells dividing to renew populations of more differentiated cells that are not self-renewing. Although ongoing cell replacement is clearly necessary for long-lived organisms, it is not obvious why tissue renewal should involve ongoing differentiation from somatic stem cells. In principle, tissues could be maintained by the self-duplication of fully functional cell types, using the same kind of “cell memory,” or direct epigenetic inheritance of cell state, that is typical of unicellular organisms [1]. Indeed, some metazoan tissues do seem to replace lost cells through such self-duplication of differentiated cells [2]. Such a simple system is both evolutionarily conserved and metabolically efficient. Instead, however, most adult tissues replace lost cells through a much more elaborate system that we call “serial differentiation.” In this system, new fully differentiated cells are not produced by the division of fully differentiated cells (which are incapable of division), but by the division of “transit” cells, or “transient amplifying cells” (TACs). One tissue may include a series of several TAC stages, each of which results from the division of the preceding stage. These cells are transient in the sense that they only proliferate for a limited amount of time before they become terminally differentiated and are eventually shed from the tissue. They amplify the proliferative potential of the somatic stem cells, because each TAC stage doubles the number of descendent cells that ultimately result from the division of a somatic stem cell [3]. Serial differentiation has been described in a variety of self-renewing tissues [4–10]. Relative to direct epigenetic inheritance of cell state by fully differentiated cells, the more elaborate system of serial differentiation presumably requires greater genetic complexity and also entails a metabolic cost in supporting the additional cells. Therefore, it would seem unlikely to have evolved unless it provided some important advantage to the organism. Here, we propose that this advantage lies in the suppression of somatic selection and thus somatic evolution.

### The Challenge of Somatic Evolution

A multicellular organism can be viewed as a population of cooperating cells. This population is subject to the same evolutionary processes as any other population undergoing reproduction, death, mutation, and competition for limiting resources. Selection within a metazoan will inevitably favor those cells that are better at reproductive competition and survival [3]. Yet, the characteristics that help cells compete effectively within the organism are generally detrimental to organismal integrity and fitness [11]. Thus, there is a fundamental conflict between selection among cells within organisms (“somatic selection”) and selection among organisms [3,11–14]. Multicellular organisms could not emerge as functional entities before organism-level selection had led to the evolution of mechanisms to suppress cell-level selection [15–18]. Cell differentiation has previously been recognized as a mechanism to control somatic evolution and its potential for carcinogenesis during development [10,19,20]. However, even in mature tissues, the combination of cell turnover and somatic mutation creates the conditions for somatic evolution. The conflict between the cellular and organismal levels of selection is exacerbated in long-lived organisms with extensive cell turnover. Humans are estimated to contain approximately 10^14^ cells with extensive cell turnover [21]. For example, each day, the small intestine and the hematopoietic system shed 10^10^ and 10^11^ cells, respectively [22,23]. Furthermore, many of the genes in a metazoan genome may function to constrain cellular competition and coordinate cellular cooperation [15]. This implies that many loss-of-function mutations may provide a competitive advantage for the mutant cell [24]. Accumulation of such somatic mutations through cell-level selection could lead to two general classes of pathology. First, diversion of cell resources into cell survival and replication and away from organismal function could impair a wide range of organismal functions, leading to general senescence [25]. Second, the shedding of restrictions on cell division and survival, if unchecked, ultimately leads to uncontrolled cell proliferation and cancer [3,20].

### The Hypothesis: Serial Differentiation As a Defense Against Somatic Evolution

Somatic evolution is inevitable given the cumulative Darwinian selection that occurs in any self-renewing population of proliferating cells, together with a supply of variation in cell fitness from somatic mutation. In multicellular organisms with substantial cell turnover, both self-renewal and cell proliferation are necessary, and the complete suppression of all mutation may not be achievable. However, it may be quite feasible through differentiation patterns to almost entirely suppress somatic selection, without which the appearance of the occasional somatic mutation is harmless to the organism. We hypothesize that this is achieved in animals by compartmentalizing self-renewing tissues such that one cell population (stem cells) undergoes self-renewal, while another (TACs) undergoes active proliferation. If no cell population combines both these necessary elements of somatic evolution, somatic evolution is thereby suppressed. If this separation is imperfect, some somatic selection may occur, but it will be greatly weakened and slowed. Consequently, we would expect to see the pathologies associated with somatic evolution to persist at some level, but primarily as ailments of old age.

We hypothesize that stem compartments are subject to little somatic evolution because stem cell populations are small and quiescent, with little proliferative activity. Populations of TACs are subject to little somatic evolution because they are not self-renewing, so that somatic Darwinian selection is not in effect: mutations conferring increased cell survival and replication do not increase in frequency within this cell population.

Here, we test our hypothesis using a simplified computational model of cell population dynamics and differentiation in a tissue or proliferative unit (e.g., an intestinal crypt). Our hypothesis requires the representation of stem cells, TACs, symmetric and asymmetric cell divisions (which may be self-renewing or differentiating), and the ability to track the fate of mutations that increase the fitness of a cell lineage. In addition to the effects of self-renewing cell divisions, we also study factors influencing the rate of somatic evolution under serial differentiation, including symmetric versus asymmetric differentiation, the number of differentiation stages, and loss-of-function mutations in differentiation pathways. The model is not designed to faithfully replicate the details of any one tissue, but rather to capture the essential dynamics relevant to our hypothesis.

## Results

### Effect of Number of Differentiation Stages

**Figure 1:**
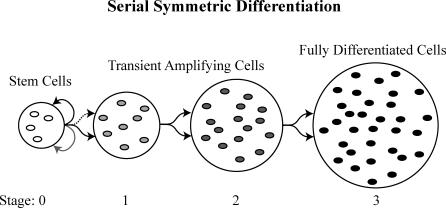
A Diagram of Serial Differentiation The series includes stem cells (stage 0, in white), TACs (in gray), and finally, terminally differentiated cells (stage 3, in black). Stem cells divide asymmetrically with one daughter rejoining the stem cell compartment and one daughter differentiating (black arrows), unless the stem cell population is below homeostatic levels, in which case both daughter cells become stem cells (gray arrow). If there is an overabundance of stem cells, both daughter cells will differentiate (dotted arrow). TACs divide symmetrically so that both daughter cells advance to the next differentiation stage. Thus, every cell division outside the stem cell compartment entails differentiation into the next downstream stage, eventually ending in the terminally differentiated cells, which are purged from the tissue (e.g., sloughing of the outer layer of the skin, or the upper cells of an intestinal crypt into the lumen of the gut).

Our first set of experiments was set in the context of serial cell differentiation, under the assumption that cell differentiation was symmetric, with both daughter cells sharing the same fate (Figure 1). We allowed somatic mutations that either increased the cell's intrinsic division rate, or reduced its intrinsic probability of death (e.g., through apoptosis), and observed the outcome of somatic selection.

#### Experiment 1A: Fate of single selfish-cell mutations.

Our first experiment used the introduction of controlled mutations (see Methods) to examine the probability of a single mutation sweeping to fixation within the cell population, as a function of the cell-level fitness advantage it conferred.

**Figure 2:**
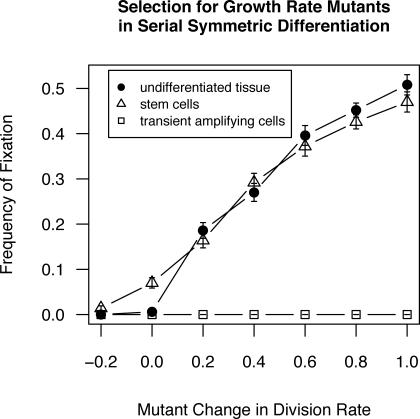
Frequency of Fixation (Mutant Reaches >90% of the Cell Population) for Mutations Affecting Cell Division Rate Frequency of fixation in nondifferentiating populations (filled circles), stem cells (triangles), and TACs (squares) with standard error bars. Because they did not vary, results for all three transient amplifying stages are pooled.

**Figure 3:**
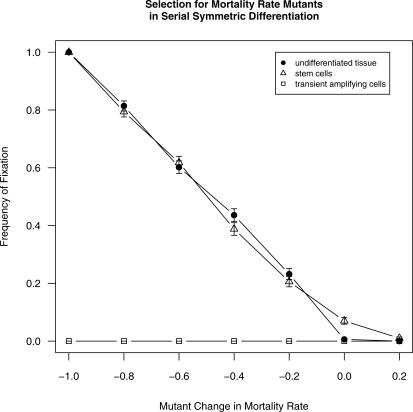
Frequency of Fixation (Mutant Reaches >90% of Cell Population) for Mutations Affecting Cell Mortality Rate Frequency of fixation when introduced into nondifferentiating populations (filled circles), stem cells (triangles), and TACs (squares). Bars show standard errors. Because they did not vary, results for all three transient amplifying stages are pooled. Values on horizontal axis show mutant cell's value of *d* relative to initial value of 0.05. Mutant values of *d* thus ranged from (0.05 − 100% = 0) to (0.05 + 20% = 0.06).

Because our model included stochastic cell mortality and replication, when a novel mutant was first introduced into a population as a single cell, it was in danger of going extinct through drift even if its intrinsic fitness was higher than that of its competitors. If it survived long enough to reproduce and establish a clone, an advantageous mutation tended eventually to spread to fixation. In nondifferentiating (self-duplicating) cell populations, mutations increasing the division rate by even a moderate degree grew to fixation with high frequency (Figure 2). Under serial differentiation, such mutations sometimes spread to fixation when introduced into stem cells. Mutations introduced into non-stem cells never reached fixation, but instead were always lost from the population, regardless of the mutant's replicative advantage (Figure 2). When mutations were introduced that increased the cell's intrinsic survival rate, the results were similar (Figure 3).

#### Experiment 1B: Accumulation of quantitative selfish-cell mutations.

Experiment 1A showed that in contrast to nondifferentiating tissues, under serial differentiation, non-stem cells were invulnerable to accumulating selfish-cell mutations that increased cell survival and replication. This suggests that somatic evolution could be suppressed by making stem cell populations so small that selection was weak relative to drift. In our model, if we assume that the sustained number of terminally differentiated cells (*K_n_*) is dictated by the needs of the organism, then the required number of stem cells (*K_0_*) can be reduced without increasing their rate of proliferation by increasing either the number of non-stem differentiation stages (*n*), or the ratio of cell numbers between subsequent differentiation stages, according to the expression

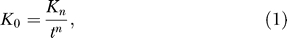
where *t* is the ratio of cell numbers between one stage and the next. Here, we assume that cell numbers double with each stage of differentiation (*t* = 2) and focus on the number of non-stem differentiation stages (*n*).

**Figure 4:**
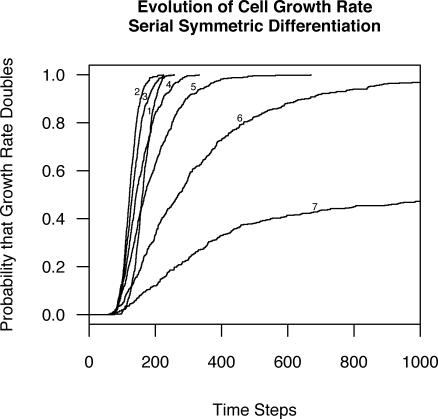
Kaplan-Meier Curves for the Probability that a Population of Cells Doubles Its Average Growth Rate as a Function of Time Each curve is labeled with the number of differentiation stages in the model (1 = self-duplication with no differentiation).

**Figure 5:**
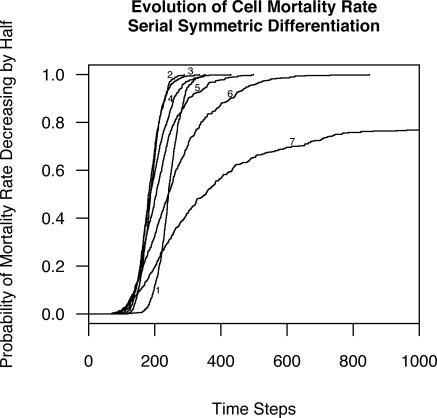
Kaplan-Meier Curves for the Probability that a Population of Cells Halves Its Average Mortality Rate as a Function of Time Each curve is labeled with the number of differentiation stages in the model.

Using the protocol of stochastic mutation (see Methods), we allowed the quantitative cell trait of either intrinsic replication rate or mortality rate to mutate at each cell division, and measured the resulting time until the average population replication rate doubled or mortality rate was cut in half. As the number of differentiation stages increased, so did the total number of cells, the number of cell divisions per time step, and the number of novel mutations arising per time step. All else being equal, these factors would increase the rate of somatic evolution. Nonetheless, as the number of differentiation stages increased, so did the waiting time before we recorded a 2-fold evolutionary change in the trait under study (Figure 4). For every differentiation stage added to the model, the relative risk (RR) of the cell population doubling its intrinsic replication rate was cut in half (Cox proportional hazard RR = 0.498, 95% CI: 0.476–0.522, *p* < 0.001). The same was true for the effect of number of cell stages on waiting time until intrinsic mortality rates were halved (RR = 0.689, 95% CI: 0.667–0.712, *p* < 0.001). The Cox regression takes into account both the time until the 2-fold change, and the fact that some simulation runs were censored at 10,000 time steps [26]. Kaplan-Meier curves illustrate this pattern for both the doubling of intrinsic replication rate (Figure 4) and the halving of intrinsic mortality rate (Figure 5).

The response to varying *n* was not linear. It may seem surprising that somatic evolution was more rapid with two cell stages (stem and fully differentiated) than with a single self-renewing cell stage (Figure 4). This is because the two-stage situation required stem cell divisions to supply replacement cells to both the stem cell compartment and the differentiated cell compartment. Thus, even though this situation involved half as many stem cells as the situation with a single self-renewing cell stage, it involved more total stem cell divisions and therefore produced cell turnover in the self-renewing stem compartment, leading to faster somatic evolution than the single-stage situation (Figures 4 and 5). Because both the number of stem cells and their replicative activity were important, the most effective suppression of somatic selection occurred with more than four non-stem stages (*n* > 4; Figure 4).

### Effect of Asymmetric Differentiation

For some cells, differentiation may be asymmetric, with one daughter cell remaining in the parental cell stage and the other further differentiating [27]. Because such asymmetric division represents a form of self-renewal by the parental stage, we hypothesized that allowing it in TACs would increase the rate of somatic evolution. To test this hypothesis, in Experiment 2 we repeated Experiment 1A, but included one treatment with asymmetric instead of symmetric differentiation in all TAC stages.

#### Experiment 2: Effect of asymmetric differentiation on somatic evolution.

Compared with symmetric differentiation, asymmetric differentiation resulted in more rapid somatic evolution. In a multivariate Cox regression controlling for number of differentiation stages, asymmetric differentiation increased the risk of intrinsic replication rate doubling (RR = 1.56, 95% CI: 1.41–1.73, *p* < 0.001).

**Figure 6:**
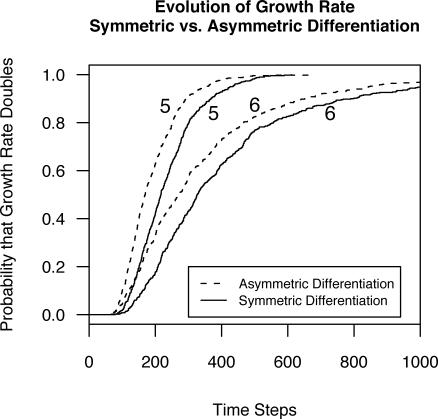
The Probability of Doubling the Population Average Growth Rate as a Function of Time, Asymmetric or Symmetric Differentiation, and Five or Six Differentiation Stages in the Models Models with asymmetric differentiation evolve more quickly and are therefore more susceptible to pathologies of somatic evolution (RR = 1.56, 95% CI: 1.41–1.73, *p* < 0.001).

In addition, as we observed with symmetric differentiation in Experiment 1B, under asymmetric differentiation, somatic evolution slowed as the number of differentiation stages increased. This was true for cell replication rate (Figure 6; Cox regression: RR = 0.547, 95% CI: 0.530–0.560, *p* < 0.001) as well as cell mortality rate (RR = 0.704, 95% CI: 0.688–0.719, *p* < 0.001).

### Self-Renewal in TAC Stages

Some published models of cell differentiation assume that non-stem cell populations are partly self-renewing (e.g., [5,28]). To study this scenario, we assumed TACs were like stem cells in that both of their daughter cells could either remain in the parental differentiation stage or proceed to the subsequent stage, depending on homeostatic signaling mechanisms that maintain an equilibrium number of cells in each stage. According to our hypothesis, such self-renewing cell populations would be more vulnerable to somatic evolution than would tissues following strict serial differentiation (Figure 1).

#### Experiment 3A: Effect of symmetric self-renewal on somatic evolution.

**Figure 7:**
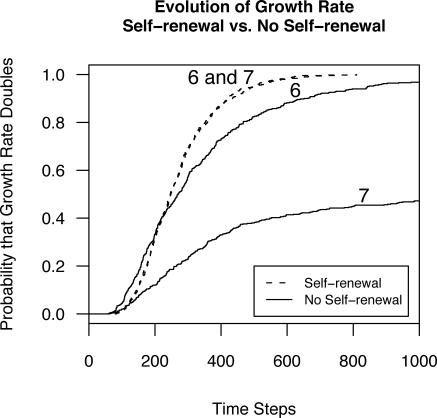
The Probability That the Average Growth Rate of the Population Doubles as a Function of Time, Number of Differentiation Stages (Six or Seven), and Self-Renewal by TACs Versus Serial Symmetric Differentiation The tissues with self-renewing TAC stages evolve more quickly than the tissues with strict serial differentiation, where, with the exception of the stem cells, both daughter cells of a mitosis differentiate into the next differentiation stage. TACs, dashed lines; serial symmetric differentiation, solid lines.

To test the hypothesis that self-renewal by TACs would accelerate somatic evolution independently of whether division was asymmetric or symmetric, we repeated Experiment 1A under the scenario of symmetric self-renewal by TACs. We observed that symmetric differentiation with self-renewal was more prone to somatic evolution than was symmetric differentiation without self-renewal (Figure 7; for doubling of intrinsic replication rate, RR = 1.86, 95% CI: 1.66–2.09, *p* < 0.001; for halving of mortality rate, RR = 1.39, 95% CI: 1.25–1.54, *p* < 0.001).

#### Experiment 3B: Fate of discrete selfish-cell mutations in self-renewing cell compartments.

To clarify the reasons for the results of experiment 3A, we again examined the fate of single selfish-cell mutations using our controlled mutation protocol, this time including a treatment of symmetric self-renewal by TACs.

**Figure 8:**
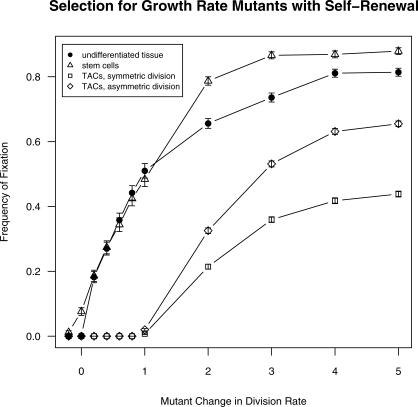
Frequency of Fixation by Mutations Affecting Cell Division in Nondifferentiating Populations, Under Serial Differentiation with Self-Renewal (*n* = 4 Non-Stem Stages) for Stem Cells, Transient Amplifying Stages Dividing Symmetrically, and Transient Amplifying Stages Dividing Asymmetrically Each data point represents at least 500 trials with standard error bars. Nondifferentiating populations, filled circles; serial differentiation with self-renewal for stem cells, triangles; transient amplifying stages dividing symmetrically, squares; transient amplifying stages dividing asymmetrically, diamonds.

**Figure 9:**
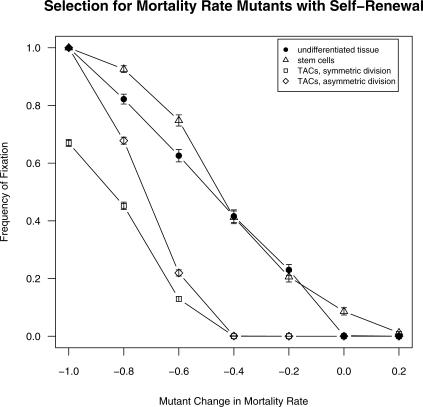
Frequency of Fixation by Mutations Affecting Cell Mortality in Nondifferentiating Populations, Under Serial Differentiation with Self-Renewal for Stem Cells, Transient Amplifying Stages Dividing Symmetrically, and Transient Amplifying Stages Dividing Asymmetrically Each data point represents 500 trials with standard error bars. Cells that cannot apoptose (mortality change = −1.0) may still differentiate into the fully differentiated stage and stop dividing. Nondifferentiating populations, filled circles; serial differentiation with self-renewal for stem cells, triangles; transient amplifying stages dividing symmetrically, squares; transient amplifying stages dividing asymmetrically, diamonds.

As predicted, allowing self-renewal by the TACs did increase fixation rates for selfish-cell mutations introduced into TACs. For example, a mutation conferring a 5-fold increase in division rate was more likely to spread to fixation when TACs underwent self-renewal then when they did not (Figure 8). This was true whether the mutation arose in a cell at stage 1 (multivariate logistic regression: odds ratio [OR] = 1.45, 95% CI: 1.41–1.49), at stage 2 (OR = 1.24, 95% CI: 1.21–1.28), or at stage 3 (OR = 1.06, 95% CI: 1.03–1.10) (*p* < 0.001 in all cases). Similarly, a mutation conferring a 5-fold decrease in mortality rate was more likely spread to fixation when TACs underwent self-renewal then when they did not (Figure 9). This was true whether the mutation arose in stage 1 (OR = 1.54, 95% CI: 1.46–1.61), stage 2 (OR = 1.49, 95% CI: 1.42–1.57), or stage 3 (OR = 1.17, 95% CI: 1.12–1.23) (*p* < 0.001 in all cases). For both types of selfish-cell mutation, the only condition that entirely prevented them from ever spreading to fixation in TACs was TAC symmetric division without self-renewal (Figures 8 and 9).

As one would expect, allowing self-renewal by the transient amplifying stages did not change fixation rates for selfish-cell mutations introduced into stem cells (multivariate logistic regression OR = 1.01, 95% CI: 0.99–1.03).

### Mutations that Disrupt Differentiation

The foregoing experiments show that serial differentiation can effectively block the spread of selfish-cell mutations that increase cell survival and replication. However, loss-of-function mutations can also affect cell differentiation itself. In this section, we investigated whether differentiation knockout mutations were positively selected, and how they affected the dynamics of cellular evolution and proliferation. We found that differentiation knockout mutations were positively selected within the cell compartment they arose in because they caused both daughter cells to remain in the parental stage. They also caused a striking increase in cell proliferation.

#### Experiment 4A: Fate of controlled differentiation knockouts.

Our first experiment in this part used our “controlled mutation” protocol to follow the fate of a knockout mutation introduced into a single cell in a TAC stage. When a single mutation was introduced, it tended to spread to fixation, except in the instances where it was lost through stochastic drift, while still very rare. If the mutant clone did not go extinct within approximately 100 time steps, it expanded exponentially to fixation.

**Figure 10:**
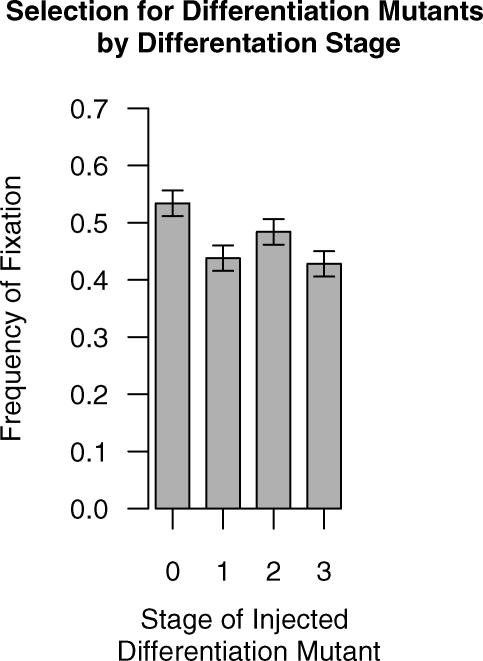
Frequency of Fixation of a Differentiation Knockout Mutant as a Function of Time The model was run for 500 timesteps to allow the cell populations to equilibrate before a mutant cell that could not differentiate was injected. Those mutant clones that did not go extinct in the next 100 timesteps expanded rapidly. Mutants injected into the stem cell compartment reached fixation more frequently than mutants injected into a TAC compartment. Each data point represents 500 trials with standard error bars.

These clonal expansions occurred despite the fact that the cells remained subject to normal feedback controls on division rate. This is because the differentiation knockout mutation interacted with the negative feedback mechanism of tissue homeostasis to eliminate normal growth inhibition. Because the nondifferentiating clone did not provide any cells to the downstream differentiation stages, the terminally differentiated cell compartment tended to fall below its initial population size. This caused proliferative stimulation of all the TACs and the stem cells, including the nondifferentiating mutant cells, due to the high division probabilities that resulted from low downstream numbers in conjunction with negative-feedback control of proliferation (Methods and Equation 4). Differentiation knockouts were selectively favored in any cell capable of division. Despite the fact that stem cells were normally capable of foregoing differentiation, differentiation knockouts were selectively favored even in this compartment (Figure 10).

#### Experiment 4B: Effect of stochastic selfish-cell mutations.

**Figure 11:**
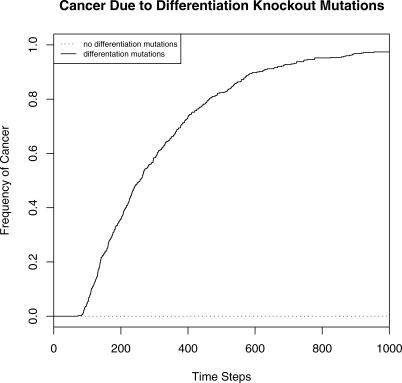
Kaplan-Meier Curves for the Time Until Exponential Cell Growth (Cancer) Were Detected Due to a Spontaneous Differentiation Knockout Mutation The solid line represents 500 trials during which differentiation loss-of-function mutations occurred at a rate of 10^−4^ per cell division. The dotted line represents the Kaplan-Meier curve for 500 trials in which no differentiation mutations were allowed. A differentiation knockout mutation can cause exponential cell population growth in any nonterminal differentiation stage driven by the feedback loops that normally maintain tissue homeostasis.

**Figure 12:**
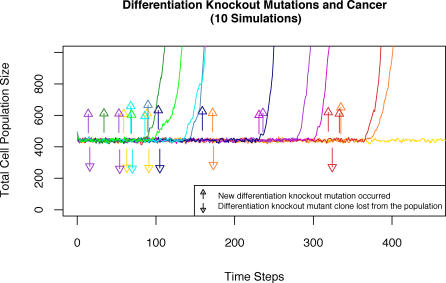
Cell Population Size in Response to Stochastic Occurrence of Differentiation Knockout Mutations The total population size for more than 400 simulation timesteps is pictured here for ten different runs in different colors. Color-matched arrows indicate when a new differentiation knockout mutation occurred and when the resulting mutant clone went to extinction. Note that the new differentiation knockout mutations (if not lost from the population) develop into cancer with a lag time of between 70 and 150 timesteps. The stochasticity of the process is illustrated in one run (in yellow) that never progressed to cancer even though it acquired and lost several differentiation knockout mutations. In each run, homeostasis of cell numbers can be seen up until the appearance of a differentiation knockout mutation.

Our second experiment in this part used our “stochastic mutation” protocol to impose a risk of differentiation knockout accompanying each cell division, at a rate of 10^−4^ per daughter cell. Under these conditions, exponential growth in the cell population (interpreted as neoplasia) developed quickly (Figure 11). Traces of cell population sizes from individual runs show that even though differentiation knockout mutations frequently occurred, most quickly went extinct due to stochastic drift (Figure 12). If they survived long enough, they eventually triggered exponential growth due to interaction with the homeostatic feedback mechanisms (Figure 12).

## Discussion

### Summary of Key Findings

We have shown that, in principle, ongoing serial cell differentiation in mature tissues can suppress cell level selection and somatic evolution. We suggest that this pattern of cell differentiation was a critical step in the evolution of large and long-lived metazoans with extensive cell turnover. Serial cell differentiation makes it possible to segregate proliferative activity and population self-renewal into different cell compartments so that no compartment possesses all the attributes necessary for somatic evolution.

Our simulation experiments confirmed our a priori prediction that self-renewal in TACs would allow rapid somatic evolution because a mutant clone can sweep to fixation within a population of such cells. In addition, they revealed that asymmetric cell divisions are one of the forms of population self-renewal that would increase the rate of somatic evolution. Symmetric cell division without self-renewal suppresses somatic evolution by causing the constant removal of non-stem mutations from the proliferating cell population. All such mutations are trapped in cell lineages that are destined to rapidly die out through terminal differentiation. Any form of self-renewal, including asymmetric cell division, disrupts this purging dynamic, allowing the sequential accumulation of multiple selfish-cell mutations.

### Implications for the Evolutionary Transition to Multicellularity

The evolution of multicellularity from preexisting unicellular life is one example of repeated events during evolution in which new kinds of biological “individuals” have emerged from collections of previously existing entities. In each of these “transitions in individuality,” selection at the level of the newly emergent individual is thought to have created mechanisms to suppress internal selection among its subunits, which would otherwise disrupt its integrity and lower its fitness [15–17]. Several such mechanisms have been proposed for the evolutionary transition to multicellular life. Buss argued for the central role of germ line segregation [15], which protects the germ line from being affected by somatic evolution within the soma. In contrast, Queller [29] emphasized the importance of a single-celled stage in the life cycle for limiting the genetic variation passed on to offspring. Both of these mechanisms may be important to mitigating long-term somatic evolution across many organismal generations.

Here, we propose another mechanism that has long been known to exist, but the functional significance of which has received little attention (but see [30]). By suppressing somatic evolution within tissues, serial differentiation may not only suppress somatic evolution across generations of multicellular organisms. In addition, it may also suppress the short-term somatic evolution that can have significant deleterious consequences within the lifespan of a single organism that is large and long-lived [31].

Cell differentiation depends upon epigenetic inheritance. Thus, our results support the suggestion of Jablonka [1,32] that epigenetic inheritance played a central role in the transition from unicellular to multicellular life by helping to control selection among the cells of the newly emergent multicellular individual. Because the epigenetic state of the genome is heritable across cell generations, somatic evolution almost certainly occurs in the epigenome as well as in the genome. Epigenetic alterations are commonly detected in cancers and are thought to often be early events in carcinogenesis [33–37]. Thus, changes labeled as somatic “mutations” in our model could also be realized by epigenetic alterations.

### Testing Our Conclusions against Empirical Data

The main purpose of this paper has been to test a hypothesis for the evolutionary origin and function of an already-familiar pattern of cell differentiation in large metazoans. Given that the existence of this pattern is not in doubt, what testable predictions follow from our hypothesis that could be used to reject or support it? Several predictions concerning the architecture of normal, healthy tissues in long-lived metazoans can be tested experimentally or even by careful observation. One such prediction is that large and proliferating (e.g., non-stem) cell populations are not expected to be self-renewing. Another prediction is that when non-stem cells divide, both daughter cells must be committed to further differentiation. Thus, cell division should be intimately tied to differentiation. Dividing without committing to differentiation is the definition of self-renewal, and we have shown that this is a risk for somatic evolution and associated pathologies.

Opportunities for testing our hypothesis arise where our predictions appear to conflict with the prevailing view of cell differentiation in some tissues. For example, it has been suggested that mouse pancreas β cells self-renew without contribution of non–insulin-producing stem cells [2]. If confirmed, this would raise questions about our results, or about how these pancreatic cell populations avoid rampant somatic evolution and accompanying pathologies. Other interpretations of the evidence are possible, however. One hypothesis proposed by Dor et al. that is consistent with our results is that not only terminally differentiated β cells, but also unipotent β stem cells and TACs, produce insulin [2]. If this is true, then serial differentiation in a cryptic form may be present even in pancreas β cells, as our hypothesis would predict.

As another empirical test, the hematopoietic system is commonly assumed to involve self-renewing TACs [5,38]. We predict that closer study will reveal these morphologically indistinguishable cells to be functionally stratified into a series of non–self-renewing TAC stages. Our prediction is that when such a TAC divides, its daughter cells are one division closer to terminal differentiation than the parental cell. This must involve some form of “counter” for mitoses such that cells that are more generations removed from their ancestral somatic stem cell in the tissue are closer to terminal differentiation than are cells that are fewer generations removed from their ancestral stem cell. This prediction is supported by the observation that, although hematopoietic cells may appear to be self-renewing stem cells based on cell-surface markers, some have limited self-renewal capacity, as is typical of TACs [39]. In solid tissues such as an intestinal crypt, the mitosis “counter” might be implemented by position in the tissue, as long as proliferating cells move up the gradient of differentiation when they divide [40]. However, for the principles we have elucidated to apply, the functional sequence of differentiation stages need not correspond directly to physical location.

Perhaps the most important empirical counterexample to our hypothesis for the suppression of somatic selection is the adaptive immune system, which contains large cell populations that are both self-renewing and actively proliferating throughout life. The apparent reason for this exception to the general rule of serial differentiation is that the adaptive immune system depends on somatic evolution though clonal selection for its effectiveness ([41], p. 15). According to our hypothesis, serial differentiation greatly slows somatic evolution in most tissues, forestalling its pathogenic consequences until old age. Because this is not true of the adaptive immune system, we would expect pathologies arising through somatic evolution to manifest at much earlier ages in these cell populations than in other tissues. Indeed, leukemia and lymphoma are unusual cancers in that they are relatively common at younger ages. Whether the adaptive immune system is also vulnerable to accelerated senescence relative to the rest of the body has not been closely investigated to our knowledge, but there are intriguing clues. The thymus is an important site of somatic selection in the adaptive immune system ([42], p. 223), and it is known to atrophy beginning at puberty ([42] p. 44). Thymectomized adult mice that received a transplanted thymus enjoyed improved immune function. However, the improvement was significantly greater when the donor was newborn versus 33 mo old ([42] p. 45). This functional decline, even before the age of 3 y, could be due either to deleterious somatic evolution or to other causes. In humans, the decline in immune competence that occurs with aging is a serious clinical concern. Aging is associated not only with declining competence but also with dysregulation of immunity, including increasing autoimmune disorders [43]. This is consistent with a possible failure to suppress inappropriate cell proliferation in the immune system, as might result from prolonged somatic evolution.

Other empirical predictions of our hypothesis can be tested with phylogenetic data. In organisms with high cell turnover, and thus great potential for somatic evolution, suppression of somatic selection through serial differentiation is especially critical. It is in such organisms that organismal selection should most strongly favor the effective but costly mechanism of DNA methylation as a means of maintaining the differentiated state in somatic cell lineages. This prediction has been met by analysis of taxonomic patterns in DNA methylation [32].

Another empirical prediction concerns body size. Whenever evolution has scaled organisms up from small and short-lived to larger and longer-lived, the potential for somatic evolution has increased [44]. Our model suggests that because somatic stem cells normally form a self-renewing cell compartment, they pose the highest carcinogenesis risk on a per-cell basis of any cells in a tissue. In our model, it is possible to increase the number of cells and the amount of cell turnover per organism without increasing the number or proliferative activity of somatic stem cells, simply by increasing the number of non-stem stages (*n*) as per Equation 1 above. This is what we would expect to see in comparisons between species with different body size. This prediction is consistent with previous theory [17,44,45] and also with data showing a lower ratio of stem to fully differentiated cells in the feline hematopoietic system relative to that of the mouse [46].

### General Medical Implications

If we are correct in our hypothesis for the control of somatic evolution through serial differentiation, it may have important medical implications. Both diseases involving uncontrolled cell proliferation (cancers), and those involving generalized loss of normal tissue function, are good candidates for conditions arising through the expression of unrestrained somatic selection. For this reason, research into the etiology of such diseases should include a focus on postembryonic patterns of cell differentiation.

If our hypothesis is correct, it may help in understanding why senescence and general loss of tissue function is a typical part of aging. Somatic evolution has been proposed as a fundamental source of senescence [25]. We have shown that serial differentiation can reduce somatic evolution, but not completely eliminate it. Some proliferation by stem cells is necessary, and self-renewal of TACs can arise by sporadic somatic mutations disrupting normal differentiation. Be slowing somatic evolution, serial differentiation may not entirely eliminate senescence but delay it until old age. This would suggest that conditions of accelerated senescence, or progeria syndromes, may result from a failure to suppress somatic evolution. In this regard, it is significant that patients with Hutchinson-Gilford progeria syndrome appear normal at birth, while the disease is usually diagnosed near the end of the second year of life after failure to thrive commences. This pattern suggests that in patients with this condition, fetal, but not postnatal, development is normal [47].

### Implications for Cancer

The conventional wisdom is that cancer begins with genetic or epigenetic lesions causing excessive cell proliferation. The results presented here suggest a more nuanced picture of the dynamics of carcinogenesis. We have shown that in the context of normal serial differentiation, a genetic lesion causing excessive division by its host non-stem cell will not result in uncontrolled cell proliferation. On the other hand, a genetic lesion that has no direct effect on division rate, but that disrupts normal cell differentiation, may quickly lead to abnormal cell proliferation.

For purposes of prevention and early detection, it is critical to understand the earliest stages in carcinogenesis. Our results may be useful in this regard. It is clear that any cell population that is both actively proliferating and self-renewing is at high risk of somatic evolution and thus of carcinogenesis. It is also clear, however, that there are two distinct ways this situation may arise. One is that a (normal) self-renewing population of stem cells may acquire mutations that increase proliferation or reduce apoptosis. Such “selfish-cell” mutations will immediately be favored by selection among stem cells, and may rapidly go to fixation within the stem cell compartment if not lost by genetic drift. This route is facilitated by any factors that increase the rate of stem cell replication, including factors that are normal in themselves, such as wound healing and cyclic growth of breast and reproductive tissues [48,49].

The second basic route to tumorigenesis begins with non-stem cells such as TACs. These compartments are normally large and proliferative but not self-renewing. Here, somatic selection among cells will not favor increased replication or survival unless preceded by an initiating mutation that blocks normal differentiation and thereby converts the resulting clone into a self-renewing population. After this initiating step, all further selfish-cell mutations will spread and accumulate through somatic selection. In contrast to the stem cell pathway, neoplasia originating in TACs is not facilitated simply by higher rates of cell turnover and proliferation. Instead, it is highly dependent on a specific class of mutations, and thus may be more stochastic and unpredictable, but also more dependent on mutagens that tend to target genes involved in cell differentiation.

It seems likely that distinguishing between these two distinctive pathways in early carcinogenesis may reconcile what could otherwise appear to be conflicting evidence about the earliest steps of tumorigenesis. Moreover, neoplasms resulting from these two different early pathways may retain persistent differences that are relevant to medical strategies for their detection and treatment. The potential importance of stem cells in tumorigenesis has received considerable attention recently, partly due to the recognition of cancer stem cells [50]. Some research models have therefore focused on the role of somatic stem cells and their differentiation patterns [27]. Our results, however, emphasize that proliferating cells with a stem-like capacity for unlimited self-renewal can potentially arise from mutations in either stem cells or TACs.

The role in tumorigenesis of normal mechanisms for tissue homeostasis has received little attention, but our results suggest this might be a fruitful avenue of research.

One intriguing result of our model is that the structure of the feedback loops that maintain tissue homeostasis can have a dramatic impact on the probability that the tissue will develop cancer. Without any redundant checks on cell proliferation in this simplified model, a differentiation knockout mutation will generate uncontrolled growth if the mutant cell is not quickly cleared by stochastic background mortality. This single mutation generates uncontrolled proliferation, though an interaction with the negative feedback controlling the production of terminally differentiated cells. When a differentiation knockout mutant arises, that cell's progeny will no longer contribute to the terminally differentiated compartment. Thus, when the terminally differentiated compartment drops below equilibrium levels, stem cells and TACs are stimulated to proliferate, including the mutant. The mutant clone will grow, taking over more of its compartment and thereby further reducing the tissue's capacity to replenish the terminally differentiated cells. A vicious cycle ensues in which the more the mutant clone grows, the more the terminally differentiated compartment signals the need for more proliferation. The result is an exponential expansion of the mutant clone. These results are similar to those of a computational model of skin in which differentiation was based on distance from the basal membrane mediated through mechanical and adhesive forces in the tissue [51]. Rashbass et al. found that disruption of the differentiation responses of the cells could lead to exponential cell growth. Of course, in a real tissue, the growth of the mutant clone would be limited by additional proliferative repression and by nutrient availability until further mutations could generate stimuli for neo-angiogenesis. Our model highlights the importance of the relatively understudied mechanisms of tissue homeostasis.

Based on our results, we predict that genetic lesions disrupting differentiation are often critical to tumor initiation. Because of the small size and low activity of somatic stem compartments, it seems unlikely that any tissue with serial differentiation would accumulate the mutations necessary for cancer unless differentiation was disrupted early in the process. Considerable evidence supports this view. For example, blocked differentiation is a frequent theme in the development of hematopoietic malignancies ([52], p. 470). Similarly, lesions in *APC,* a gene involved in differentiation in crypts of the intestine [53], are considered “gatekeeper” lesions that initiate colonic adenomatous polyps and are necessary for the future development of colorectal cancer [54]. In another tissue, recent genome-wide analyses have shown that most alterations in acute lymphoblastic leukemia target the B cell differentiation pathways [55].

It is worth noting that many of the cell characteristics considered to be hallmarks of cancer are examples of “selfish-cell” traits that are favored by somatic selection when it is operating. These include traits that reduce intrinsic mortality rate, such as evasion of apoptosis, as well as traits that increase intrinsic division rate, such as self-sufficiency in growth signals and insensitivity to antigrowth signals [56]. When we understand that somatic selection is the underlying process driving carcinogenesis, it is clearly no coincidence that “most if not all cancers have acquired the same set of functional capabilities during their development, albeit though various mechanistic strategies” ([56] p. 59). Furthermore, when we understand the central role that disruption of normal differentiation plays in allowing somatic selection, this suggests that early loss of differentiation may eventually be recognized as one of the most fundamental, and earliest to appear, of the hallmarks of cancer. If true, this could point toward important directions in using genetic tests to screen for cancer, or even sporadic cancer risk, before the first directly observable symptoms appear.

One active area of cancer research involves the use of chemotherapeutic agents that act by promoting cell differentiation [57]. The feedback loops that maintain tissue homeostasis are likely to modulate the efficacy of these differentiation agents. It may be important to interrupt those feedback loops so as to prevent the cancer cells from increasing their proliferation rate to compensate for cells lost to differentiation [10,20,28,38,55,58–61].

Some of the ideas we have explored in this study have been raised previously in the specific context of carcinogenesis. Cairns proposed that the elaborate system of somatic stem cells, TACs, and terminally differentiated cells in a gastrointestinal crypt is an adaptation to suppress cancer [20]. Mutations that occur in the TACs of a crypt are destined to be sloughed off in a matter of days [6]. Only the self-renewing stem cell population, or mutant cells that no longer differentiate properly, are vulnerable to mutations that may establish an expanding clone and become locally fixed. Somatic stem cells are typically quiescent and few in number [6,62]. These traits may be organismal adaptations that both reduce the frequency of somatic mutations and limit the role of somatic selection relative to genetic drift in stem cell populations [30,63]. We suggest that the structure of serial differentiation may be a general principle for the suppression of somatic evolution and thus neoplasia not just in gastrointestinal crypts but in all tissues with extensive cell proliferation. Even in the less physically structured hematopoietic system, serial differentiation may serve to limit somatic evolution.

Charlton [64] proposed that somatic evolution underlies the pathogies of both cancer and senescence, but did not address how somatic evolution might have been avoided in people without these pathologies. The idea of somatic evolution has also been explored in some detail in the cancer biology literature: previous agent-based models of carcinogenesis have been used to explore theories of the clonal evolution that drives neoplastic progression [65–69]. Mathematical models have also been used (e.g., [27,70]). While these previous studies have all highlighted the detrimental effects of somatic evolution, they have not focused on the question of what normally suppresses somatic evolution, and thus have not completely explained what key turning points cause healthy tissue to become neoplastic.

Kirkland has developed a model of differentiation in the hematopoietic system that is conceptually similar to ours. In Kirkland's model, cell stages are not discrete, but rather, “stemness” is represented as a continuous variable [71]. A probability density function determines the stemness of daughter cells. Although “stemness” was represented as a continuous variable in Kirkland's model, and as a discrete variable in our model, the same principles apply in both, and both reached similar conclusions: if daughter cells can be as undifferentiated as the parent cell, then self-renewal occurs and the tissue is vulnerable to somatic evolution. Only tissues in which the daughter cells are more differentiated than the parental cell are protected from somatic evolution. The same concept also applies to theories of stem cell niches where extrinsic properties of the microenvironment determine the differentiation state of cells [72]. In this case, differentiation is determined by migration, and we would predict that non-stem cells should migrate out of but not into the stem cell niche.

Tomlinson and Bodmer also developed a similar model, with self-renewing compartments of stem cells, TACs, and differentiated cells [28], which was extended to include homeostatic feedback mechanisms [70,73]. In this model, failures of apoptosis or differentiation led either to clonal expansion to higher equilibrium cell numbers (benign tumors) or to extended exponential growth of the cell population (neoplasm). Nowak et al. showed that a linear model of a crypt, with a single stem cell and asymmetric division at all cell stages, limits somatic evolution and slows progression to cancer [30]. Frank et al. also analyzed a model of a crypt in which stem cells and TACs could have different mutation and division rates [73]. They found that differences in mutation rates between the cell compartments changed the optimal proportion of cell divisions between the compartments to minimize somatic evolution [73].

Several previous authors have proposed that the failure of cell differentiation plays an important role in tumorigenesis [10,20,28,58–60]. We have expanded on this idea by showing how cell differentiation prevents the onset of cell proliferation through controlling cells' selective environment and thereby suppressing somatic evolution. Similarly, several previous authors have recognized that somatic evolution occurs and is probably central to neoplastic progression [3,38,74–76], and that tradeoffs in evolution and the selective pressure of cancer may have shaped multicellular genomes and bodies [11–14,31]. Here, we have shown how somatic evolution is normally controlled, and how that control can break down during the events preceding tumorigenesis. Finally, we have shown that the loss of differentiation interacts dramatically with the feedback loops that maintain tissue homeostasis and may lead to clonal expansion and carcinogenesis.

## Methods

### The model.

To investigate the role of cell differentiation in somatic evolution, we developed a simplified model of the evolutionary dynamics of cells within a tissue of an adult organism. This model consists of a set of assumptions about the behavior and population dynamics of cells within tissues, which we embodied in an agent-based computer simulation. The source code for the computational model is freely available from the authors upon request. A detailed description of the model's assumptions and algorithms follows.

### Representation of cells.

Each cell was represented by three heritable characteristics: intrinsic replication rate, intrinsic mortality rate, and whether or not it was capable of differentiation upon division. A fourth cell characteristic was its current differentiation stage. When a cell underwent mitosis, it ceased to exist, and was replaced by two daughter cells. Each daughter cell inherited the first three of the intrinsic characteristics listed above. Their current differentiation stage was typically incremented from that of the parent cell. (Instead, the current differentiation stage was directly inherited without being incremented in various scenarios of self-renewing compartments described below, including stem cells, self-renewing TACs, and mutant TACs with differentiation knockouts.) Intrinsic growth and mortality rates were modeled as quantitative traits. Capacity to differentiate was a binary value representing either the functionality of differentiation pathways in the cell, or their disruption by mutation. Current differentiation stage was an integer (*i*) ranging from 0 (for a stem cell) to *n* (a terminally differentiated cell). A model parameter determined the total number of non-stem cell stages (*n*). The control of cell division and differentiation is further described below (Tissue homeostasis).

At the start of a simulation run, all cells had the same intrinsic growth rate (*r*) and mortality rate (*d*), set by parameters (Table 1). All initial cells were assumed to be capable of differentiation until a somatic mutation disrupted that capacity.

**Table 1:**
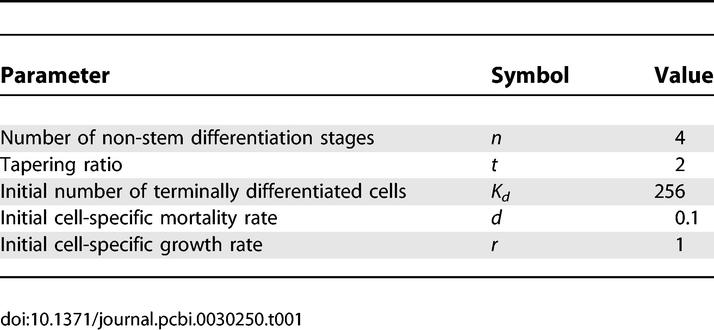
Standard Parameter Values

During each timestep, every cell had the opportunity to divide or to die, with the stochastic probability of each determined by its intrinsic values of *r* and *d*, respectively. If a cell was capable of differentiation, then immediately upon dividing, its daughter cells had the opportunity to advance to the next differentiation stage. The control of cell division and differentiation is further described below (Tissue homeostasis).

### The cell population.

The model represented a population of cells constituting a tissue or proliferative unit. This population included cells in a series of differentiation stages, indexed by *i*, ranging from stem cells (*i* = 0), continuing through 0 or more transient amplifying stages (0 < *i* < *n*), and ending with terminally differentiated cells (*i* = *n*).

The initial number of cells in each differentiation stage increased from one stage to the next by a factor of *t* (for tapering ratio), where *t* = *K_i_*_+1_ / *K_i_*. Thus, the initial number of cells in each differentiation stage *i* (*K_i_*) was determined by a combination of the parameters for the initial number of terminally differentiated cells (*K_n_*) and the tapering ratio (*t*) such that:

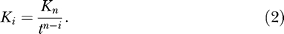
Thus, the total number of cells of all stages in the modeled cell population was:

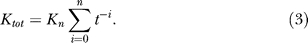



### Nondifferentiating tissues.

For comparison to tissues organized by serial differentiation, we also modeled hypothetical nondifferentiating tissues in which all cells were self-duplicating with unlimited replicative potential. These cells were also capable of performing the work of the organ for the benefit of the organism. For valid comparisons, it was important to use the same number of functional cells for differentiating versus nondifferentiating tissues. We assumed that all cells were functional in the hypothetical nondifferentiating tissues, but that under serial differentiation, only the terminally differentiated cells were functional. We therefore compared a nondifferentiating tissue containing a given initial total number of cells (*K*) against a serial differentiation series ending with that same number of terminally differentiated cells (*K_n_* = *K*), but containing a greater number of cells in total (*K_tot_* from Equation 3 above).

### Tissue homeostasis.

Presumably, homeostatic mechanisms maintain the appropriate size of cell populations in the various tissues of metazoans. Cell proliferation must be stimulated when needed, and suppressed when not needed. Little is known about the homeostatic mechanisms in most tissues [6]. In our model, we assumed that cell division was regulated by extrinsic microenvironmental signals such as competition for limited growth factors [9,77] or end product inhibition, by end products generated by the terminally differentiated cells (as has been shown in the hematopoietic system [78]), so that cell division is responsive to the number of terminally differentiated cells. In our model, the probability of non-stem cell division (*p_i_* for 0 < *i* ≤ *n*) followed a logistic function:

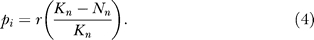
where *r* was the cell-specific intrinsic growth rate and *K_n_* and *N_n_* were the initial and current number of terminally differentiated cells, respectively. The probability of division was truncated at the limits of 0 and 1. The effect was to maintain the number of terminally differentiated cells close to the initial number. These feedback loops are a simplified representation of the roles of stromal cells, cytokines, morphostats [10], and cell-to-cell contact in regulating cell proliferation to maintain tissue homeostasis. The result of implementing these rules in our model was that initial cell numbers were maintained as an equilibrium between cell production and cell loss to terminal differentiation and death (Figure 12).

For any TAC stage *i*, (0 < *i* < n) cells entered the stage through division and differentiation from stage *i* − 1, and exited the stage through cell mortality as well as division and differentiation to stage *i* + 1.

In addition to non-stem cells, the number of stem cells must also be regulated [6] in order to replenish stem cell losses due to apoptosis and cytotoxic exposures and thereby preserve the integrity of the entire proliferative unit. We modeled the probability of stem cell division (*p*_0_) as the sum of stimulation from both the stem and the terminally differentiated compartments:

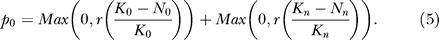
where *K*_0_ was the initial number of stem cells and *N*_0_ was the current number of stem cells. The use of the maximum function here prevented suppression of cell division due to an overabundance of one cell type from interfering with the replenishment of the other cell type.

When a stem cell divided, each daughter cell differentiated into the next stage (TAC stage 1) if and only if the stem cell population was at or above its initial population size (*N_0_* ≥ *K_0_*).

In our simulation, cells differentiated only immediately subsequent to mitosis (in the same timestep), though this rule could represent differentiation at any time between mitosis and the next cell cycle. When a stem cell divided, both daughter cells remained stem cells if the stem cells were below their initial number (*N*_0_ < *K*_0_). Otherwise, one daughter cell differentiated into the first transient amplifying stage (stage 1), while the other became a stem cell (stage 0) [79–81].

The differentiation of TACs was modeled in two different ways for comparison. Under symmetric differentiation (Experiments 1A and 1B), when TACs divided, both daughter cells differentiated into the next stage in the series. Under asymmetric differentiation in TAC stages (Experiment 1C), one daughter cell remained in the same cell stage as the parent and the other daughter cell progressed to the next cell stage. To model self-renewal by TACs (Effect of Asymmetric Differentiation), we had them behave like stem cells in that daughter cells from each stage differentiated into the next stage if and only if their own stage was at or above its initial population size (*N_i_* ≥ *K_i_*). Unless otherwise noted, parameter values for all simulation runs approximated values from the gastrointestinal crypt literature (Table 1) [6].

### Experiments.

In our experiments, we introduced somatic mutations of the three heritable cell characters: intrinsic replication rate, intrinsic mortality rate, and capacity for differentiation upon division. The evolutionary outcomes we measured were the average values of quantitative traits (intrinsic replication or mortality rate), the frequencies of discrete mutant alleles for differentiation ability, or changes in total cell population size.

To study the effects of mutations affecting rates of replication and mortality, we carried out two types of experiments, using either a controlled mutation of a single cell at a time, or stochastic mutation of all dividing cells.

*Controlled mutations.* In controlled mutation experiments, we turned off stochastic mutation, let the model equilibrate for 500 timesteps, and then introduced a single mutant cell with an altered rate of intrinsic replication or mortality, or (in Mutations that Disrupt Differentiation), with heritable loss of normal differentiation ability. We ran the model for 10,000 timesteps, or stopped it sooner if and when the mutant clone either went extinct or to fixation. To reduce run time and reduce the extreme stochasticity of time to complete fixation with drift, we used as a proxy for fixation a mutant allele reaching a frequency of >90% of the cell population. Because our model did not include any frequency-dependent fitness effects, it was safe to assume that any mutation that increased from an initial low frequency to >90% would eventually have gone to fixation given sufficient time.

We introduced mutations of varying magnitudes into different differentiation stages, and each case was tested at least 100 times with different random number seeds.

*Stochastic mutations.* In experiments with stochastic mutation, we let the growth or mortality rates mutate as follows: upon each cell division, the growth or mortality rates of the daughter cells were changed to represent the quantitative effects of mutations caused by DNA replication errors during cell division. At cell division, each daughter cell inherited the parental cell's quantitative trait multiplied by a normally distributed random variable with mean 1 and standard deviation 0.05. Thus, stochastic mutations could either increase or decrease these traits, with equal probability.

In experiments with stochastic mutation, we stopped each simulation run when either the average intrinsic growth rate doubled or the mortality rate was halved from the initial rates, or after 10,000 timesteps, whichever came first. We varied the number of cell stages in the model from just one stage (nondifferentiating tissue) to seven cell stages (stem cells, terminally differentiated cells, and five intervening TAC stages).

We also used mutation experiments to study the effects of differentiation knockout mutations (see Mutations that Disrupt Differentiation). In Experiment 4, we allowed stochastic mutations to disrupt differentiation pathways at a rate of 10^−4^ mutations per cell division. This mutation was heritable upon cell division, so that it prevented further differentiation in the entire resulting clone. We stopped the simulation run when either the clock reached 10,000 timesteps or the total cell number reached ten times the initial level, which we interpreted as the initiation of a neoplasm. Each experiment was replicated at least 100 times.

## Abbreviations

OR: odds ratio
RR: relative risk
TAC: transient amplifying cell
